# Parents’ Experience in Children’s Friendship Training Programme for Their Children with Autism Spectrum Disorder: A Qualitative Inquiry

**DOI:** 10.3390/children8090763

**Published:** 2021-08-31

**Authors:** Sing Yee Ong, Samsilah Roslan, Nor Aniza Ahmad, Ahmad Fauzi Mohd Ayub, Lee Ping Chen, Sahar Mohammed Taresh

**Affiliations:** 1 Faculty of Educational Studies, Universiti Putra Malaysia, Seri Kembangan 43400, Malaysia; syee718@gmail.com (S.Y.O.); nor_aniza@upm.edu.my (N.A.A.); afmy@upm.edu.my (A.F.M.A.); sahartaresh30@gmail.com (S.M.T.); 2 Department of Psychology, School of Medicine, International Medical University, Kuala Lumpur 57000, Malaysia; Nicole_Chen@imu.edu.my; 3 Department of Kindergarten, Faculty of Education, Taiz University, Taiz 6803, Yemen

**Keywords:** friendship, training, parents, autism spectrum disorder (ASD), Malaysia

## Abstract

Background: Children’s Friendship Training (CFT) is a parent-assisted intervention programme that introduces children to basic sets of social rules to help them understand social contexts with specific guidance from their parents. It has been reported in several empirical studies that the friendship skills of children with autism spectrum disorder were enhanced after participating in CFT. However, previous studies only focused on the effectiveness of the training without exploring it from the parent’s perspective. As such, the objective of this study is to highlight the parents’ experience in assisting in the implementation of CFT. Purpose: To explore the parents’ experiences with autism spectrum disorder (ASD) in CFT and examine the experiences using the CFT as a theoretical framework. Methodology: In this study, eight parents and their school-aged children with ASD participated in 12 CFT sessions. Upon completing the CFT, the parents participated in a focus group interview. The interview session was video recorded and transcribed with the parents’ consent. Thematic analysis was employed in analysing the collected data as outlined in six different phases. Results: The generated data revealed the similarities and differences in parents’ experiences in the CFT. The current study has identified four main themes: (1) fear and resistance; (2) awareness, learning, and adjustment; (3) change is hard; and (4) identifying support. Conclusions: The findings highlighted the processes that these parents experienced and encountered while attending the CFT programme, it is important to consider these processes based on how they might impact the effectiveness of the programme. The programme’s effectiveness is reliant on the ability to work closely with parents to understand their challenges and explore the type of support they need. This study has analysed the crucial factors that provide an overview of parents’ encounters in their participation in CFT.

## 1. Introduction 

Autism spectrum disorder (ASD) is a neurodevelopmental disorder in which social communication, social interaction, social imagination, and intellectual flexibility are all impaired [1,2]. There is no exception in Malaysia since many children live with ASD, and many parents struggle to care for them. Unfortunately, the current recorded children with ASD in Malaysia do not reflect the actual number because not all cases are registered under one roof. In an article on “Special Effort Needed for Special Needs Kids” by [3], the statistics indicated that at least one in 600 children in Malaysia had been diagnosed with ASD. Based on this ratio, from a total of 7.6 million children under 14 in 2019 (current population estimates, Malaysia, 2018–2019, 2019), 1,770,800 of these children have ASD, while others remain undiagnosed. This statistic means that more attention is needed to understand the population with ASD better and provide the support needed by their parents. 

It is without a doubt, that parents go through many challenges in dealing with their children with ASD [4]. Often, parents, the key caretaker of children with ASD, encounter issues in their psychological well-being (worries, anxiety, emotional exhaustion) and physical well-being (easily being hurt physically) [4,5]. They also experience a high level of financial and social burden [5,6]. Other than that, they also have concern about the quality of intervention services provided to their children [6].

Since social skills deficits are one of the notable challenges that children with ASD encounter, the Children’s Friendship Training (CFT) programme was established [7]. CFT is among the few empirically established peer functioning interventions designed specifically for the participation of school-aged children, involving parents in the intervention. The parents and children must attend 12 sessions concurrently as stated in CFT, the evidence-based manualised intervention [8].

This parent-assisted intervention programme introduces children with ASD to basic social rules to help them understand the social context with specific guidance from their parents. Several empirical studies have proven that CFT can improve friendship skills among children with ASD.

CFT was developed by [7] for the UCLA Parenting and Children’s Friendship Training Program. It is a manualised parent-assisted intervention group, which involves teaching and developing the children’s friendship skills with specific guidance from their parents [9]. Several empirical studies have proven CFT to improve friendship skills and social skills; thus, reducing loneliness among children with ASD [9,10].

To our knowledge, previous studies have only focused on the effectiveness of CFT without exploring it from the parent’s perspective. Furthermore, parental relationship closeness is mentioned as the main parameter that influences the success of CFT [11]. Many children have been diagnosed with ASD, yet there are limited resources available for their families, especially parents, who need to care for their children with special needs. As previously mentioned, children with ASD often face difficulty in their social interactions with others. As such, it is crucial to include parents in training their children in social communication. Parents are significant in their children’s intervention as they are the key people who spend most of their time taking care of them [12]. Their involvement is one of the factors that could influence the outcome of the intervention [13,14]. They can share valuable information with the professionals [15] and be recognised as competent interventionists [16]. Frankel and Myatt [7] saw the potential of integrating parents into this training programme to help children manage their social interactions [7]. Adapting this idea from Ladd et al. [17]. In this training programme, parents play the role of integrating, assisting, supervising, and providing solutions to their children in their friendship building process.

However, the parents’ views and experiences have often been neglected by CFT management and are unknown by researchers, especially in different cultural contexts such as Malaysia. In the setting of Southeast Asia, parents see themselves as vital members of an inclusive and interconnected whole in their family, job, country, or religious group [18]. Previous research (e.g., [19,20,21]) has shown that parents of ASD children from Southeast Asian communities are more likely to feel affiliate stigma and have more depressive symptoms than parents from Western/European societies. There is also evidence that stigma emerges differently in collectivist, group-based societies than in individualist cultures (e.g., [5]). In Malaysia, Yeo and Lu [22] discovered that a cultural component predicted parental stress and psychological suffering among children with ASD. As mentioned by Daley et al. [23] and Freeth et al. [24], it is essential to include cultural aspects while investigating CFT from the parents’ perspective. This study’s objective is to highlight Malaysian parents’ experiences in assisting the implementation of CFT by attending 12 sessions with their children with ASD, since their views and experiences have often not been given importance. 

## 2. Methods 

This study is part of a larger mixed-methods study that investigates the impact of the CFT on social skills among children with ASD conducted by [11]. This larger study has indicated improved social skills and friendship quality after participating in CFT [11]. The qualitative phase of this larger study is the current focus of this study. A qualitative interpretive design was chosen because it was suitable to explore and understand the parents’ encounters and perspectives [25]. This study used a qualitative approach to provide a complete picture of the participants’ “lived experience” by explaining the primary cause and motivation behind their participation in CFT. The authors had obtained approval from the Medical Research Ethics Committee Malaysia (MREC) (NMRR-17-321-334530 IIR) and University Putra Malaysia Ethics Committee to conduct this study. Informed consent was obtained before the first CFT session was conducted.

### 2.1. Participants 

Purposive sampling was used to recruit *n* = 8 parents. Eligible participants were chosen among those who have children with the following inclusion criteria: (a) diagnosed with ASD; (b) currently schooling; (c) can understand and speak English; (d) can differentiate the winning and losing concept; (e) have issues with making friends; and (f) attended at least nine sessions of CFT with their children. Parents were excluded if they attended less than nine CFT sessions with their children and could not understand and speak English.

### 2.2. Data Collection

The semi-structured interview sessions were conducted upon the completion of the CFT, and parents were made aware that they needed to participate in a focus group interview session. They were invited to a face-to-face focus group interview, whereby each interview session would last approximately 1.5 to 2 h. This study conducted two focus group interview sessions to record the experiences of these parents in implementing CFT with their children. The participants were interviewed using open-ended questions to explore their feelings, thoughts, and opinions regarding their experiences with CFT with flexibility. The interview questions included, questions such as “What were the issues or challenges during your participation in CFT?”, and “What were your experiences in CFT?”. The interview sessions were video recorded and transcribed. Basic demographic data were also collected, classified into two groups, namely, the parents’ details and the child’s details, as stated in Table 1 and Table 2. 

### 2.3. Data Analysis

Thematic analysis by [26] (p. 36) is employed to interpret the data collected in six different phases: (1) knowing the data, (2) generalisation of initial codes, (3) searching for themes, (4) reviewing themes, (5) naming and definition, and (6) producing the report. 

Firstly, the first author read through the original transcript produced from the recorded videos word by word. This was to establish the initial code and the code sequence from the transcript. The initial code may be in one word or sentence. Then, the first and fifth researchers repeatedly read the original transcript independently using the codes as proposed by [27] to establish the emerging themes relevant to the study’s objective. Next, all researchers were involved in grouping the initials codes to reach a consensus in finalising the concluding themes. Finally, the final report was prepared. Triangulation and peer check were applied to ensure the trustworthiness of the data. All researchers were present at the time of peer check (peer debriefing), and after the majority of the researchers agreed on one theme, this step was repeated for all the themes. There was 100% agreement from all authors regarding the finalised themes.

Triangulation was used by comparing the data obtained from the focus group interview sessions to identify the research themes. Peer check was also used, with the researchers coming together to analyse the data and reach a consensus on the identified themes. These methods were used to minimise the personal biases of the researchers, which could affect the research outcomes. 

## 3. Findings

**Figure 1:**
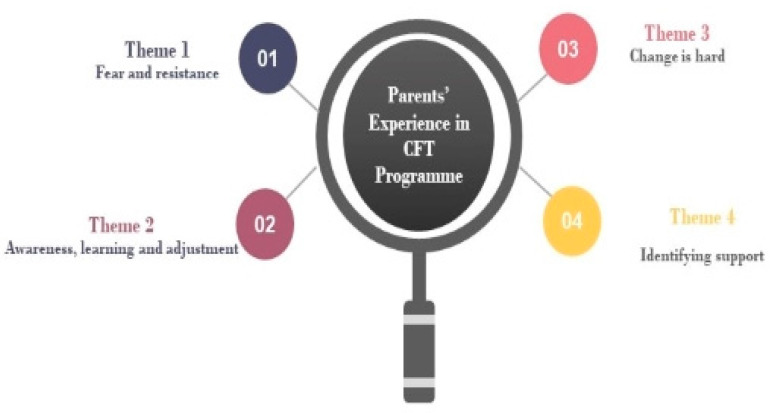
Parents’ experience in CFT Programme.

The generated data exhibited both similarities and differences in the experiences among parents who participated in the CFT programme. Figure 1 shows the four main themes identified in this study: (1) fear and resistance; (2) awareness, learning, and adjustment; (3) change is hard; and (4) identifying support.

Theme 1: Fear and resistance

The first observed theme was fear and resistance. This theme was noticeable among parents who shared their emotional stage, whereby they claimed that they were fearful, felt self-blame, and were even ignorant of the child’s condition during the initial stage of their participation in CFT. Most of these parents feared making mistakes, as described by three parents (P3, P5, and P8), who shared their fear during the interview. They were afraid that their child would cause many behavioural issues when they were with their peers, which would later require the parents to solve them; indeed, they feared just bringing their child out. Parent P5 was worried that her son embarrassed her and his peers, thereby causing stress to the parent.

“Last time, it was so tense that I’ve been holding his hand with other kids playing. Make sure he well behaved up to that level. I make sure that he doesn’t embarrass like he doesn’t make mistakes like, too rough, not playing together, or running away. So, I always said, do it together….”

Parent P3 was aware of her fear of taking her son out to the park to play with other children, as she wanted to avoid complaints about her son’s behaviour.

“Because before this, avoid Marcus to play with, I mean, avoid him to play with others some strangers. Because I don’t want any problem to occur. You know, it’s a lot of friends complain about him. Say something that make him upset. So, I think the biggest changes is on me….”

She expressed in tears,

“Even the time to playground also, the time to play with kids.”

Parent P8 was also afraid whenever she needed to take her daughter out to meet other children.

“I think I observe more whenever she with her friends, right? Normally I go anywhere, or her cousins come, or when she’s out when I take her. Very honestly to tell right, whenever I take her out, I will also have, there’s a fear in me.”

Several parents even talked about their ignorance of their child’s condition.

Parent P5 admitted her ignorance of her son’s condition and would only instruct him to do things without listening to him and thinking he was still young.

“For myself, I think the biggest thing that I have for pre and post is that I used to be opposite. Either I don’t care, I just put him aside, or I come and this one cannot, that one cannot….”

She further expressed that the training programme has helped her gain insight into herself because she was not trying hard enough to reach out to her child. She stated,

“So, you may ask me, any ‘Aha’ moment on my child. I previously, it’s not that I don’t have time, but I just ignore my son, maybe half of it, is like that ar. I have to be blamed of….”

Theme 2: Awareness, learning, and adjustment

The second theme concerned the parents’ awareness, learning, and adjustments throughout the 12 CFT sessions.

*Awareness*

In the aspect of awareness, several participants talked about their awareness, which came about after they began participating in the training. Parent P7 claimed that he began to realise that he and his wife were inclined to treat his son as a “special child” and needed to give more attention to his “special trait,” which limited his interaction with others.

“So, first thing is the; we are now more aware the need to have peer group for interaction all that. Last time, we said, ok, he interacts with friends; after school, he interacts with us. So, for example, swimming classes is one on one. He used to have Karate classes one on one. Interact part; he does it in school. I had WhatsApp, my wife, just now, me and my wife talking about the karate class with others instead of private karate class. So, one of thing I realised is that, besides school, everything else is one on one, one on one. The reason why we doing is because he is special kid. He needs more attention. Instead of putting him into a swimming class with more kids, we put one on one, we put him one on one, like karate. Because of this, we are kinda limiting his interaction.”

Parent P4 was aware that she needed to help her child make friends naturally and in a relaxed way. She described, 

“Ya, we need to do it in a more relax manner, in a more natural manner. It is really, you know, play only. So, when people, when my son, later on, ask his friend, whatever new friend to come in. Oh, go to Isaac house’s is very fun one, so we want to create that kind of concept to the friends, so he has got more friends. Because he is introvert, he cannot influence. We cannot influence others, and we only influence with the circle of what we can. So, this is how I feel, and that is what we’re going to do after the program.”

The parents also shared their awareness about their children. Parent P5 and parent P6 confessed that they did not stress about their child’s progress in socialising with others, participating in outdoor play, and two-way communication. Parent P5 used to think that playing at the park was unimportant, as she would rather stay at home and do her house chores. 

“Ya. To be at the park. Last time I think it’s more important to be at home because I need to cook.”

Parent P6 realised that he had neglected to teach his daughter how to practise her social skills since he focused only on her academic skills growing up. He noticed this when he was answering the questionnaire.

“Fill in that time, the way I realised that, this is the important point must you get. But a lot of time this kind of point that, because usually, we start, very small time, I try to do social, social then more and more I found that academic part lacked. Teacher complaint writing, reading, everything. Then ah, the attention more go to writing, reading, doing Maths, spend more time on this kind of academic things. The play date I long time I never emphasise already.”

During the CFT sessions, two parents (P7 and P8) realised that the communication between them and their respective children was merely a one-way communication instead of two-way communication. Parent P7 stated,

“Second thing was in this course we realised that we actually never had a conversation with Vishal. In the sense that it was always one way, Vishal do this, Vishal have you done this, what was it, just we asking him and he replying…ahh and a conversation is he also have to ask questions.”

*Learning*

It was a learning process for the parents during the CFT. And all the parents acknowledged they had acquired new knowledge in participating in CFT sessions. They appreciated all the new knowledge and committed to applying this knowledge while caring for their children. This section presents the new knowledge gained by these parents. 

Parent P2 learnt that she needed to teach her son how to interact with his peers as the parent. She stated,

“I also learnt. So, not just to bring, let him join. Then, we also have to guide. This is very important. To guide him. Tell him what to do, make, making a friend. That’s very important.” 

Parent P3 took the courage to learn to be assertive when teaching and guiding her son in completing the tasks in these 12 sessions. 

“So, I think the biggest change is on me. So, I am now step forward to… (parent P3 wanting to cry), yes, guide him.”

Parent P4 also noticed that she preferred to take the time and step back to guide him step by step and made relevant adjustments to help him learn and practise. She did not want to rush him to complete the tasks given, as she believed that human beings learnt through their five senses. 

“I found that you know, I do not want to rush things, I don’t want to do it because for the sake of doing it and eh we, we have our own timetable and also our work, I’m not full-time mum, so I want to appreciate every single detail that you coach into more, you know, into more use so that can carry out on your own later on.”

Parent P5 also shared that she practised two-way communication consistently. 

“After the programme, you have to maybe find time for your child, talk to him more, even though he no response, doesn’t matter. Every day, do the same thing, continuously the same thing. Then, he may have change la, that’s I found…”

“So now it’s like I learn to see from far…Now I let go a lot, I let go and help him to see. I also learning at the same time. Not only him, I learn a lot more, to know how to help him, and he also slowly seeing I, how I accepting him, and he start to behave better because I don’t hold him so much. He feels more comfortable that I’m not forcing him to behave how he should behave.” 

Parent P7 and his wife have been learning to ‘push’ their child to join in with social activities and prompt him to ask questions in developing two-way communication. 

“Yes. So, we are here. We are pushing him to slip in, go you’ve got to play with that group, so he’ll go slip in, do 1, 2 min come back out again. Because I don’t think he shares the interest. But we keep on pushing him to join, join, join. If not, he is more than happy to sit at the corner or play by himself. But we have to push him to be more social.”

Parent P8 learnt to allow her daughter to express her opinion and emotions. She was assertive to play her role to observe, intervene, and encourage her daughter during the practice. 

“As a parent for me…is always I use to ask her the question and one of your classes you said she should ask me question so that part helped me and I start letting her, that’s why she starting talking more. Talking more on what she feels, her opinions, always I’m telling her what to do, so it turned the other way around.”

*Adjustment*

Lastly, there was also the process of adjustment for the parents in CFT. All parents described their effort in adjusting to CFT. 

Four parents (P1, P5, P6, and P7) made time adjustments to practise their newly acquired skills in their daily routine. For instance, Parent P5 scheduled daily communication with her son as part of the routine and would spend the time accompanying her son while he was doing his homework assignment. 

“…So, I think talking to him a day today is very much more important.”

“…But after starting the programme, he basically, I just say that, if I, I’ll bring you the park, but I need 15 min to prepare to go out, to make sure I turned off the stove and all that, he will sit on his bike, waiting out there. The gate opened, waiting, until I...”

Parent P7 had also adjusted his schedule by using his phone alarm to remind him to spend more time talking to his son. 

“I think the most important one is the everyday conversation. Everyday 15-min conversation. I have alarmed my phone every day. Everyday 9 o’clock…”

Two parents (P5 and P7) described the self-adjustment they made, where they approached other parents to offer playdate invitations and made the outgroup call. For example, parent P7 and his wife tried their best to reach out to people they did not know for playdates and outgroup calls, even though it was challenging. 

“The outgroup playdates, the outgroup calls and playdates, we need to go and find those who are not in contact. I think that is more difficult part. The playdate also getting people to come over.”

Two more parents (P2 and P7) had also adjusted their approach in helping their children make friends. Parent P2 wanted to engage his son in exploring the socialising opportunities when participating in different societies. 

“Even after this session also, we need to look for a society that he can join. Various groups of friends.” 

Parent P7 has arranged for certain classes to be a group session instead of a one-to-one session to create an after-school peer time for their child.

“So, I just WhatsApp-ing we need to create more outside school on peer time…” 

“So, right now, we are like, never mind whether he is special or not, let him interact. So, we are planning to do a karate class. He is already into the swimming class; we plan it to be in a group session…” 

Instead of parents going through coaching and teaching their child alone, parent P3 adjusted her way and worked together with her son as a team in CFT. She would reach out to her contact, and he would remind her of session learning and homework completion. 

“…And then the outgroup call, is a, have to try a lot and he also always remind me that you have to complete the homework for me. Because teacher will ask, does he done the homework. So, he say, make sure, make sure the homework done.”

“…Like Marcus always asked me, ‘Mummy, you finished your class. What you learn today? Did you learn anything, how to teach the kid? How…’ He will ask that way. So, I think because we also maybe know the gap, the lacking between their, yeah…so, maybe he also know we need to learn….”

Fully acceptance of who their children were was the adjustment made by two parents (P5 and P8) in CFT. For instance, parent P5 accepted her son for who he is, and this acceptance has improved their relationship to the point where her son felt more comfortable being with her. 

“What I learn is to let go, let him be who he is, for Isaac. I accept him more.” 

“…I also learning at the same time. Not only him, I learn a lot more, to know how to help him, and he also slowly seeing I, how I accepting him, and he start to behave better because I don’t hold him so much. He feel more comfortable that I’m not forcing him to behave how he should behave.” 

Parent P6 would always have his daughter tag along with him 24/7, but he has recently adjusted to create personal space for both himself and his daughter. It was a relief, and he could have more of his own time.

“…like eh, how about Saturday you bring her to your house play…so I get relieved a bit ar. If not, sometimes my schedule is working, Evelyn, working, Evelyn.”

“Ya. Follow me everywhere. Last time…then the. Now at least I got her playdates.”

“…So, at least you know? Turn-taking, I also feel got a bit of my own time.”

Theme 3: Change is hard

This section aims to report the challenges that the parents faced during the process of changing, which included the issue of time commitment, relationship closeness, limited resources, parents’ challenges, parents’ perception and priorities, and the children’s condition during the sessions. 

First, four out of eight parents (P1, P2, P5, and P6) highlighted that time commitment was the utmost challenge. Parents and children (both participants and guests) were engaged in busy daily schedules. For instance, parent P1′s son’s tight schedule was preventing him from participating in the ‘slipping in’ exercise consistently. 

“Emm, my difficulty is actually finding the time to bring Dayan to the playground for slipping in. Because I think, one time from my part, and also time for him. Because he has school 7.30 am to 1 pm, and then 2.30 pm to 5.30 pm. He is tired already. So, I think, I think that’s one thing hard for us….” 

Meanwhile, three parents (P2, P5, and P6) expressed their frustration and disappointment because their invited potential guest(s) were busy. For instance, Parent P2 voiced her disappointment when the invited parents declined their playdate invitation because both the parents and their child had busy schedules. 

“…Some of them is like busy. They said busy, weekday busy, busy with study. Weekend of tuition, a lot of activities for them. Is no time for kid to play, to come out the house, just to play even for two hours. Even parents also very busy.”

Secondly, five parents (P3, P4, P5, P6, and P7) asserted that their kinship with the invited parent would influence their decision to participate in the outgroup call and playdate session. 

Parents were uncomfortable approaching parents whom they were unfamiliar with. Even though parent P5 was close with the neighbourhood children, she was hesitant to approach their parents to make playdate invitations, as she was unfamiliar with them. 

“I can’t invite the neighbour kids to house, to the house to playdate because I don’t know their parents. I only see the kids, and the kids all know me very well because I always be there for, ah ya, and then I call them by name, they will call me aunty. I didn’t see you yesterday. You promised you will bring your son. So, I don’t see the parents. My household are quite safe for children all come out by themselves. So, the parents are not there. I know that which house, taken I go ding dong, your son come to my house. I know them la, but I cannot do that.” 

Third, the parents lacked the resources to get their homework assignments completed. Two parents (P1 and P7) claimed that they were out of the contact list. For instance, Parent P1 has exhausted her contacts, and so has her son, which made it difficult to proceed with making outgroup calls and playdates. 

“…The other one is finding, finding someone to call for the outgroup call and also for the playdate. …just the problem is finding someone for the outgroup call, then later the playdate. So, right now, when I asked Dayan so who to call, and then there is “err…give me time, give me time”. Actually, he is out of options. Kind of. He doesn’t have anyone that he feels like he wants to call. That’s one of the biggest; those are the biggest problems that I face.” 

Parent P1 did not want her son to join the naughty children in her neighbourhood, and sometimes when they went over to a playground, they could not locate the right age group of children for her son to practise his slipping in skill. 

“Plus, you say about the community, like the neighbours’ children. My neighbourhood is quite dodgy, haha, so I don’t think I don’t want him to mix around with the kids.”

“… Because sometimes, ar, sometimes, err, sometimes we go to the playground, but when we go to the playground, sometimes we cannot find a group of kids where Dayan can slip in.”

Next, these parents had to play a supervisory role to guide, teach, and monitor their children while completing homework assignments during the 12 sessions, when concurrently, they also had to juggle their multiple personal roles in life between the sessions. Parent P4 described her struggle to attend to her younger child, take care of her elderly parents, change her job, and move into a new house, which took up a lot of her time. 

“I have another small kid, very attention-seeking. Then we have a house we wanted to go in. We are staying with the parents, a lot of people, a lot of problems I would find. Then, I’m changing job, so it’s the time…”

Parents’ perception of their child’s progress in CFT and their priorities could impact the child’s progress. Parents can make choices to set their own pace to complete the practices during CFT. Even though the weekly module has planned out the activities, parent P4 felt that she did not want to rush to complete the practice.

“Yes! We parents, for myself, I found that you know, I do not want to rush things, I don’t want to do it because for the sake of doing it and eh we, we have our own timetable and also our work, I’m not full-time mum, so I want to appreciate every single detail that you coach into more…” 

Lastly, the parents had the opinion that the *children’s* condition could impact their progress in CFT. Four parents described their children’s condition as self-sufficient, shy, acting too soon, and having different learning styles. Both parent P5 and parent P7 described their child as an easily satisfied person who would sometimes neglect their guest(s) during the playdate session. 

Parent P5: “…He is very self-sufficient person, he just plays on his own things, and he sometimes forgot to bring the emotion of the guest. Whether is interesting for you or just merely what he wants to play, then he wants to play car; he wants to play car. So, that’s why he is ignorant of other people …”

Parent P7: “…But we keep on pushing him to join, join, join. If not, he more than happy to sit on the corner or play by himself…”

Theme 4: Identifying support

The final theme we observed was the ability of the parents to identify support or resources in completing the weekly homework assignment after every CFT session. These parents would seek a helping hand from their social network, participate actively in the CFT parent group discussion, and request professionals to practise with their children in their initiatives. Most of these parents engaged people they knew to complete their weekly homework assignments, as described by parents P1, P4, and P5. 

Parent P1 contacted her friends and parents she knew. 

“ …Because normally at least for the 4 of 5 kids that we call throughout my friends’ sons. The other are his ex-classmates from Permata Kurnia. So, among us, the parents, we know each other…” 

Meanwhile, Parent P5 contacted her son’s current schoolmate and kindergarten parents. 

“…All the help that I got was actually from the mothers in a chat group or kindergarten. The one that I actually got to know from the current school…”

Parent P4 even engaged her son’s class teacher to help seek out the potential invited child for practice. The class teacher proactively spent the time to identify suitable candidates and informed the child’s parent(s) for consent to participate in the homework assignment. 

“…So, I get my teacher to help, so the teacher really did a great job, like in term of, the teacher already draw and write an official letter, give it to his friends, pick 15 friends that he say I want to have this, this, this, this, this. So, the teacher actually drafts an official letter to the parents, ask the parents to go and sign and give the contact number to me before Chinese New Year.” 

“Teacher spend time, you know, really, in the class, and then ask, ask, ask, which are the friends you want to invite. So, the teacher selects 15 students….”

Then, parent P5, a full-time housewife herself, got her husband and her daughter to help her out with her son’s homework assignments.

“…now basically I just send my daughter. You go observe your brother; I need to cook. You go observe your brother see whether he play in group. Does he slip in and join. And then about half an hour later, I will go there, and I observe. So, I see him play until the end.”

“I’m trying to get my husband to understand the same page. I’m still doing it, to be honest, and he is also learning from me to let go.”

Some parents would actively engage themselves in weekly group sessions. Parent P5 admitted that she gained many ideas and strategies, which she found helpful in completing the homework assignment. 

“…Also listening from the group is very important. I learn from the group that this is the way, then I try it out, and I found that, eh, work, it works…I think that sharing of the sessions is very good ar, everything. Then I get tips and apply to myself.” 

These parents have initiated promoting the importance of making friends, which led them to form a playdate group. Parent P6 actively promoted the idea of making friends and its importance among other parents with special children. Subsequently, the invited parents and children became more committed to conducting playdate sessions during CFT. 

“…Then, playdates sometimes, I got invite the special child. Then, I know the other parents and all that, so I got highlight that this kind of things very important…”

He managed to get the other parents to take turns conducting the playgroup, which indirectly helped him complete the homework assignment.

“…So, now start to I pass to Evelyn, like eh, how about Saturday you bring her to your house play…” 

“…At least, because I got convey this to another, to my friend, like that. She also agrees, so this week I do playgroup. Next week, I do another. So at least you know? Turn-taking…”

Lastly, some parents agreed that they needed professional help to help them coach their children in improving friendship skills. Parent P3 admitted that she needed guidance from the professionals to help her guide and teach her son to build friendship skills. Her son had also reminded her of the gap. 

“Almost there, the professional help, I think is the most important. Like, the sessions like this, I mean, the parents is the first to have special kids, I mean the, you don’t know, because you never learn this kind of knowledge before. So, I think professional help for the parents is also important. I mean how to handle, how to teach. Like Marcus always asked me, ‘Mummy, you finished your class. What you learn today? Did you learn anything, how to teach the kid? How…’ He will ask that way. So, I think because we also maybe know the gap, the lacking between their, yeah. So, maybe he also know we need to learn. Because sometimes we need to know how, sometimes, how to help them…”

## 4. Discussion 

The goal of this study was to explore parents’ experiences assisting with CFT implementation for their children with ASD. The data revealed that the parents had diverse experiences, which could affect the efficiency of CFT. During the examination of this study, four significant themes emerged: fear and resistance; awareness, learning, and adjustment; change is hard, and identifying support. The study’s findings and their ramifications were addressed in light of the existing literature. Our research intended to add to the body of knowledge by delving deeper into the experiences that parents had when implementing CFT sessions.

We found that the three main themes found in this study—namely fear and resistance; awareness, learning, and adjustment; and identifying support—are similar to Carnall’s five-phase coping cycle, namely, “Denial”, “Defence”, “Discarding”, “Adaptation”, and “Internalization” [28,29].

First, “denial” is a stage where the individuals release their fear and anxiety, followed by resistance when they first encounter a change in the process [28,29]. Second, “defence” is a stage where the individuals begin to portray actions of protecting themselves while at the same time looking for self-adjusting strategies to cope with the new reality in their current comfort zone [28,29]. These stages are very similar to the first theme found in this study: fear and resistance. During this phase, parents would express their fear and ignorance of their children’s condition and even engage in self-blame. This was the initial stage the parents went through before they addressed their children’s social skills. 

Third, “discarding” is a stage where the individuals try to get rid of the old method of doing things and start applying new methods or creating new strategies in their actions [17,18]. Fourth, “adaptation” is when the individuals adjust themselves to fit into the new situation and look for alternatives to accomplish their work [28,29]. This stage is similarly described in our second theme of awareness, learning, and adjustment. The parents’ gained insight about themselves and their children in CFT. They learnt to inculcate a positive attitude to mentor their children while learning new skills during CFT. These parents made several adjustments to reach out and create opportunities for them and their children to explore and practise newly learnt skills in CFT. They were able to change their perceptions and attitude to complete the assignments and practise self-care.

For the final stage of Carnall’s Coping Cycle Model, “internalization” is when individuals adopt and adapt the new ways and integrate them into their unique strategies to complete their tasks [28,29]. This stage similarly describes the theme of identifying support in this study, where the parents self-initiated, sought professional help and engaged in different resources (teachers, friends, and family members) to complete their daily homework assignments in CFT.

The parents were dealing with a variety of issues throughout the transition and development of new skills, which hampered the efficiency of CFT. First, both parents (participants or guests) or both parties’ children were pressed for time to commit to the CFT because their schedules were always full. Both studies by [30] and [31] supported the time constraint problem. It is a problem that parents of special needs children in Malaysia and the United States often confront. Time constraints were indicated by Tully et al. (2017) as one of the hurdles to a father (*n* = 1001) participating in early childhood intervention in the United States [32]. According to [33], one of the challenges that parents face when supporting their children’s participation in the physical environment, in general, is a lack of time [33].

Second, relationship closeness was highlighted among the parents involved in the CFT, as it could influence the willingness of the guest(s) to participate in the CFT. Close relationships between both parents’ participants and parents of the guests, or between both parties’ children, in their opinion, would help smooth out playdate appointments and complete the weekly practice. This practice speaks to Malaysia’s collectivist culture [34]. They see themselves as an integral part of a larger whole, inextricably linked to their family, colleagues, country, or religion [18,35]. They are more comfortable socialising within their ingroup or people they know [18]. This mindset might be the reason for the difficulty faced by the parents in CFT when approaching other parents to make playdate appointments. 

Third, parents in CFT described how their multiple roles had distracted their focus in CFT. Focus is an important characteristic for individuals in the collectivist culture, stating that their actions are determined by norms, roles, and goals [18]. This trait may explain the difficulties encountered by the parents in CFT, who had to juggle multiple roles while caring for their spouse, parents, and child daily. They needed to focus on fulfilling the tasks and responsibilities given to them.

Finally, the findings revealed that these children’s schedules were jam-packed with after-school activities and that the focus on academic-related activities was affecting their weekly CFT homework. This demonstrated that the parents placed a high value on their children’s academic performance. This mindset is mirrored in East Asian culture, where parents value their children’s ability to compete with others [30]. This demonstrates the various values practised by family members in the macro-system, which can impact the functioning of special needs children in the intervention, as proposed by Bronfenbrenner’s Ecological Framework [36].

The findings reported by [33] support how the parents in CFT sought assistance. Based on a review of 14 studies, they concluded that when supporting their children’s participation in various settings, parents can use four strategies: “networking”, “educating”, “advocating”, and “creating opportunities” (school and home). The parents who participated in CFT initiated playdate sessions, raised awareness among other parents, and built relationships with people around them (teachers, family members, other parents) to complete homework assignments; thus, they were “networking”, “educating”, and “advocating.” These findings are consistent with the findings of 11 interviews with parents of children with ASD [15,31], in which these parents raised awareness and advocated for overcoming their barriers. CFT parents have also adjusted to provide better opportunities for their children in their practice (“creating opportunities”).

The parents in CFT would seek help from their informal support system (other known parents and family members), and they would also seek assistance from their functional and formal support system (CFT facilitators and schools). They have expressed their appreciation towards the CFT parents’ group discussion, providing them with alternatives to completing CFT practices. This is in line with the sharing of parents reported by [15], who stated that support groups do help in information sharing and inspirational support. It is also important to note that they needed to learn from the experts to apply the learnt skills with their children in each session. This agrees with the results stating that parents tend to seek advice from knowledgeable practitioners in managing their children [15]. 

## 5. Limitations

There are several important limitations to take into consideration in this study. The first is the generalisability of the results in this study since it involved only a small number of samples within the Klang Valley, Malaysia. Therefore, the results might indicate a narrow viewpoint of parents’ experiences in CFT. It is suggested that future studies explore parents’ experiences in CFT with a larger number of samples, which could also include different parts of Malaysia, especially the rural areas. 

The second limitation is the data collected focused largely 75% on the mothers’ views and less 25% on the father’s views. This may create a bias towards the results obtained in the study.

The process of collecting data and analysing data appeared to be the third limitation of this study. Due to limited staffing in this study, the first author, the interventionist in the CFT, also had to conduct the interview. This dual relationship may have affected the outcome of this semi-structural interview. The parents may also have felt that they were required to give positive feedback on CFT. To reduce this possibility, the researcher had introduced the purpose of the interview to the parents and welcomed any positive and negative sharing of their experience. The researcher had also shown acceptance of any answer given by these parents. Furthermore, the researcher was the only one to interpret the data, influencing the study results. The researcher engaged with the experts to discuss the coding and finalise the themes to reduce such bias. The final limitation of the present study is the short period of the CFT programme and the inability to observe and study parents’ changing perceptions. Thus, the researchers suggest a longitudinal study to assess it from a developmental perspective over an extended period. 

## 6. Implication 

Several potential implications were deduced from the findings detailed in this paper. First, the themes processed in this study (fear and resistance, awareness, learning, and adjustment) indicated the psychological process of the parents in CFT. This finding should prepare future parents that their role is not restricted solely to mentoring and coaching, as proposed in the CFT [7], but they will also learn more about themselves during the 12 sessions.

Second, the themes’ relatedness with Carnall’s coping cycle model provides additional insight towards understanding parents’ involvement in ASD treatment. Previous research has focused on parents’ roles [12], their experiences with the intervention [32,37], and the barriers and challenges they went through [38,39,40,41]. However, in-depth discussions on parents’ psychological processes have not been highlighted in these studies. Thus, this study proposes that the psychological well-being of parents who participated in interventions needs to be investigated in the future.

Third, the findings have provided important information for professionals to support the parents’ involvement in CFT. We have several recommendations for professionals interested in conducting CFT. First and foremost, instead of informing the parents a session ahead on weekly assignment details, as proposed in the CFT module [9], parents should be provided with homework assignment details before the CFT sessions begin. During a CFT session, the trainer should offer support for the inner process that the parents might be going through in terms of their emotions, perceptions, and thoughts for all 12 CFT parental sessions. Next, the trainer needs to address problems that the parents might be facing through detailed discussions, modelling, and locating of suitable resources (such as identifying suitable play places and how to reach the potential playdate locations) to help them create more effective and practical opportunities to complete their weekly practice. The utmost concern is the psychological well-being of the parents, who need to be supported along the three months to complete the CFT. A collaborative relationship should exist between the clinicians and the parents [42]; such a relationship would help improve the effectiveness of the intervention towards helping their children. 

Fourth, CFT needs the community’s participation (the school and the neighbourhood). The outcome of this study could offer enlightenment to the barriers faced by these parents and the type of support they needed to have their children with ASD be included in the community. The results have clearly shown what these parents have gone through and what support was needed. 

Last but not least, Malaysia practices collectivist culture [34], and CFT is created based on the individualism culture. The multicultural aspects in CFT are highlighted as part of the factors impacting CFT. Therefore, the practitioners have to be culturally sensitive when they conduct the CFT [43]. It is also crucial to investigate the multicultural aspects in intervention for children with special needs. Thus, more research on this aspect is needed in the future.

## 7. Conclusion 

In conclusion, the parents’ experiences who took part in the CFT intervention are critical for future reference. This study has provided insight into the psychological process, the difficulties encountered, and the adjustments made by the parents to complete the CFT practices in the Malaysian context. The similarities between the discovered themes and Carnall’s coping cycle model assist practitioners in comprehending the psychological process that the parents went through during CFT. 

These experiences provide a better understanding of what would benefit parents participating in parent-assisted therapies [37,44], and their participation is thought to be likely to improve the intervention’s outcome [12]. The findings also show that parents employed various methods to help their children while taking part in the CFT intervention. In particular, in Malaysia, they experienced different challenges in social and physical environments, due to its collectivist culture.

Researchers, practitioners, service providers, and policymakers must address parental engagement in the intervention indefinitely. They must also be culturally sensitive to encourage parent participation in the intervention, which would ultimately help their children with ASD.

## Figures and Tables

**Table 1:** Demographic Characteristics of Parents.

Parent’s Response	Parent’s Job	Parent’s Race	Number of Children
Mother (P1)	Professional	Malay	2
Mother (P2)	Professional	Chinese	1
Mother (P3)	Housewife	Others	2
Mother (P4)	Professional	Chinese	2
Mother (P5)	Housewife	Chinese	2
Father (P6)	Professional	Chinese	3
Father (P7)	Professional	Indian	2
Mother (P8)	Professional	Indian	2

**Table 2:** Demographic Characteristics of Parents’ Children.

Child’s Age	N (%)	Social Communication Disorder	N(%)
7	4 (50)	Difficulties in interpersonal communication	
8	4 (50)	Mild	1 (12.5)
**Child’s Gender**		Moderate	7 (87.5)
Male	6 (75)	Difficulties in social and initiation responses	
Female	2 (25)	Mild	5 (62.5)
**Child’s Family Type**		Moderate	3 (37.5)
Two parents	7 (87.5)	Social interaction skill deficits	
Single parent	1 (12.5)	Mild	2 (25)
**Child’s School Type**		Moderate	6 (75)
Government school	4 (50)	Pragmatic Language Impairment	
International primary school	2 (25)	Receptive language disorder	
Home- school centre	2 (25)	Moderate	5 (62.5)
**Child’s Diagnosis**		Severe	3 (37.5)
ASD	5 (62.5)	Expressive language disorder	
Asperger’s Syndrome	1 (12.5)	Mild	3 (37.5)
ASD, ADHD	1 (12.5)	Moderate	5 (62.5)
ASD, ADD	1 (12.5)	Written expressive disorder	
		Mild	3 (37.5)
		Moderate	5 (62.5)

Note: ASD = Autism spectrum disorder; ADD = Attention deficit disorder; ADHD = Attention deficit hyperactivity disorder.
